# Conus Medullaris Teratoma in an Adolescent: A Case Report and Literature Review

**DOI:** 10.7759/cureus.88758

**Published:** 2025-07-25

**Authors:** Zakariae Benyaich, Farouk Hajhouji, Mohamed Lmejjati

**Affiliations:** 1 Neurological Surgery, University Hospital of Agadir, Faculty of Medicine and Pharmacy, Ibn Zohr University, Agadir, MAR; 2 Neurological Surgery, Centre Hospitalier Universitaire Mohammed VI University, Faculty of Medicine and Pharmacy, Cadi Ayyad University, Marrakech, MAR

**Keywords:** adolescent, conus medullaris, mri, spinal cord tumors, surgical excision, teratoma

## Abstract

Teratomas are benign germ cell tumors that typically develop outside their usual anatomical origin. Their location in the spinal cord is exceedingly rare. Here, we report the case of a 12-year-old female adolescent who presented with a history of persistent lower back pain and progressive paraplegia. Spinal MRI revealed a well-circumscribed hyperintense lesion with a fat component at the level of the conus medullaris. The patient underwent a T12-L2 laminectomy, and a subtotal resection of the lesion was performed due to its strong adherence to neural structures. Histopathological analysis confirmed a mature teratoma. The patient exhibited marked neurological improvement during follow-up. Although total tumor excision is the preferred approach, subtotal resection is a viable alternative in cases where the tumor capsule is tightly adherent to neural tissue, given the low recurrence rates reported in the literature.

## Introduction

Teratomas are germ cell tumors that contain tissue from all three embryonic layers, namely, ectoderm, mesoderm, and endoderm [1]. Depending on their degree of differentiation, they can be classified as mature, immature, or malignant [2]. These tumors most commonly occur in the sacrococcygeal region, gonads, mediastinum, and cervicofacial areas [1,2]. Their presence in the central nervous system is rare, with a predilection for the pineal region [3]. Intramedullary teratomas are extremely uncommon, accounting for only 0.2-0.5% of all spinal cord tumors [2,4]. To our knowledge, only five pediatric cases of conus medullaris teratoma have been reported in the literature [1,2,5-7]. This report presents a sixth case in a child and offers a comprehensive review of the relevant literature, focusing on clinical presentation, imaging features, management strategies, and prognosis.

## Case presentation

Clinical presentation

A 12-year-old previously healthy female adolescent was admitted with a 10-month history of persistent lower back pain, progressively worsening bilateral leg weakness, urinary incontinence, and constipation over the past six weeks. Neurological examination revealed a grade 4/5 paraparesis. The tone was normal, but reflexes were hyperactive at the knees and diminished at the ankles bilaterally, with an equivocal plantar response. Sensory testing showed decreased pinprick and light touch sensation below the level of L4 and a saddle hypoesthesia, while proprioception remained intact. No cutaneous abnormalities were present, and there were no signs of occult spinal dysraphism.

Radiological findings

**Figure 1:**
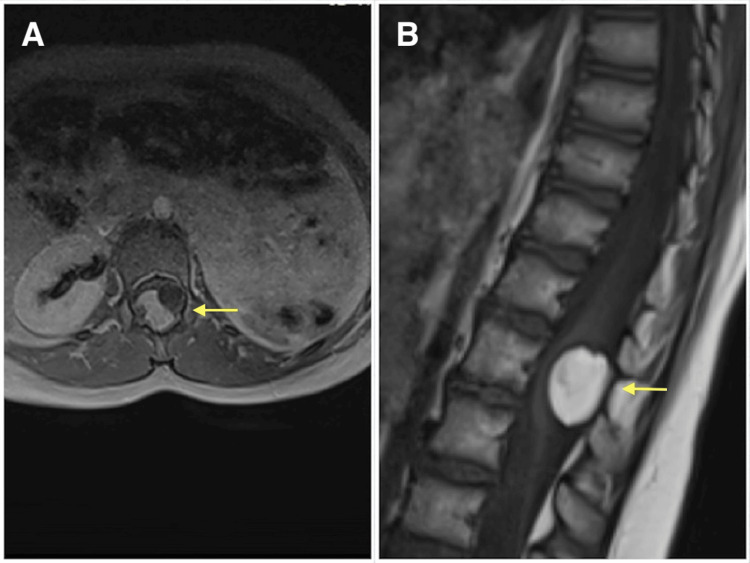
Axial (A) and sagittal (B) T1-weighted MRI scans of the spine showing a well-defined, hyperintense, intradural lesion with a nodular hypointense component at L1 (yellow arrow).

**Figure 2:**
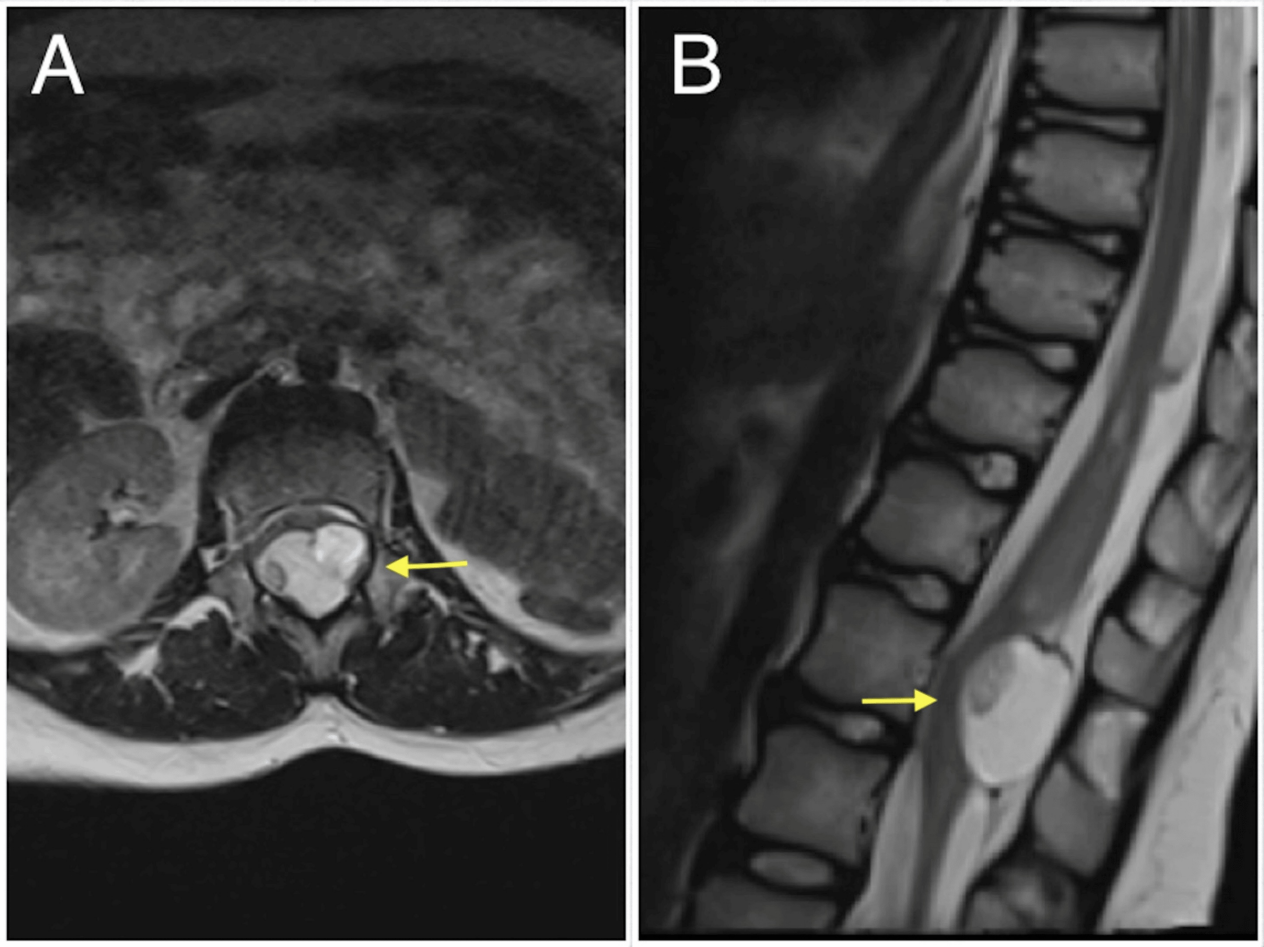
(A) Axial and (B) sagittal T2-weighted MRI scans of the spine showing a hyperintense cystic lesion at the level of the conus medullaris (yellow arrow) with a small nodular isointense area inside.

**Figure 3:**
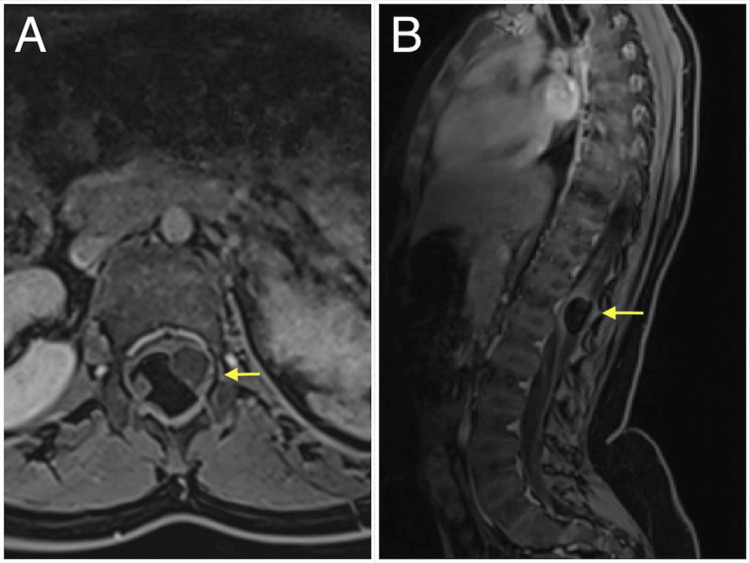
(A) Axial and (B) sagittal post-contrast, fat-suppressed, T1-weighted MRI scans showing a marked hypo-signal of fat component with mild peripheral enhancement (yellow arrow).

Spinal MRI revealed a well-defined intradural lesion in the conus medullaris at the level of L1, measuring 17 × 25 mm. The lesion was heterogenous and appeared predominantly hyperintense with hypointense components on T1-weighted images (Figure 1), and hyperintense on T2-weighted sequences (Figure 2). Post-contrast fat suppression sequences confirmed the presence of fatty tissue within the mass and demonstrated mild peripheral enhancement (Figure 3).

Therapeutic intervention

**Figure 4:**
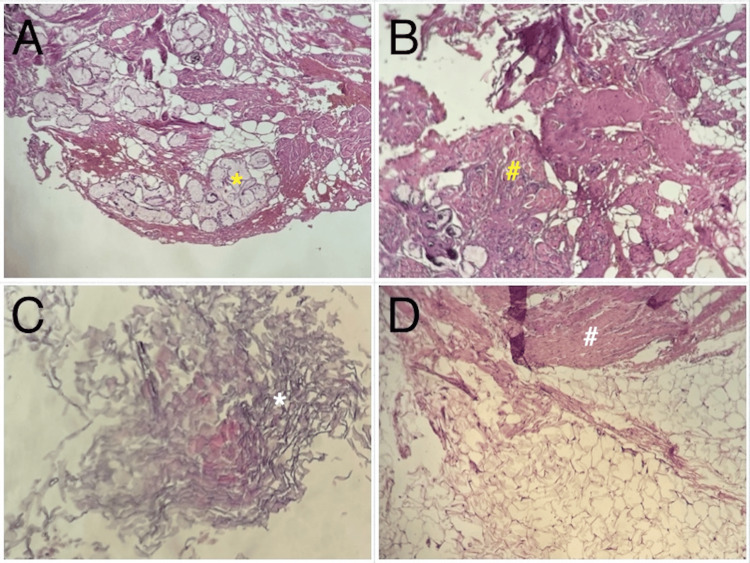
Microphotographs of the histopathological slides of the lesion demonstrating multiple mature components. (A) Fibroadipose tissue with mucin-producing glands (yellow star). (B) Peripheral nerve elements (yellow square). (C) Keratin fibers (white star). (D) Adipose tissue containing mature muscle cells (white square).

She underwent a planned C-arm-guided bilateral laminectomy at T12-L1 under general anesthesia in a prone position. Intraoperative neurophysiological monitoring was not available. A midline durotomy was performed under a surgical microscope, revealing a mass occupying the thecal sac. Upon resecting the dorsal fatty tissue, a thick capsule was encountered. When incised, the tumor contained mucous material, bony fragments, and dark hair. The contents were carefully extracted using biopsy forceps, and tissue samples were sent for histopathological analysis. Upon further inspection of the tumor cavity, a small calcified component was identified on the ventral aspect and removed using a microdissector. The dorsal portion of the tumor capsule was resected with microsurgical scissors, while the ventral portion, which was tightly adherent to neural structures, was coagulated using low-power bipolar electrocoagulation and left in place to minimize the risk of neurological damage. Approximately 95% of the tumor was successfully excised. The dura mater was then meticulously closed to ensure a watertight seal. Histopathological examination confirmed a mature teratoma composed of fully differentiated tissue components (Figure 4), with no evidence of immature or malignant elements.

Follow-up and outcomes

The patient had an uneventful postoperative recovery and was discharged three days after surgery. At the three-month follow-up, significant improvements were observed in back pain, neurological deficits, and urinary function. Motor power in the lower limbs improved from 4/5 to 5/5, reflexes normalized, and hypoesthesia resolved. The patient continued to experience occasional episodes of urinary urgency at the last follow-up. A follow-up spinal MRI is scheduled at one year postoperatively to assess for recurrence.

## Discussion

Spinal teratomas are rare lesions that can be classified as intramedullary or extramedullary, with true intramedullary teratomas being exceedingly uncommon [2,8]. These tumors typically contain elements derived from all three germ cell layers, namely, ectoderm, mesoderm, and endoderm [9,10]. However, histopathological identification of all three layers may not always be possible unless the entire specimen is analyzed [8,10]. These tumors can be associated with congenital spinal anomalies, such as spinal dysraphism or split cord malformations [2,5,11]. Although intramedullary spinal teratomas are uncommon, recent case series have identified the conus medullaris as one of the most frequently affected sites [4,8,12]. Nevertheless, to our knowledge, only five pediatric cases of conus medullaris teratoma have been reported in the literature to date [1,2,5-7] (Table 1).

**Table 1:** Summary of reported pediatric cases of conus medullaris teratomas.

Authors	Year	Age (years)/Sex	Clinical presentation	Associated anomalies	Extent of removal	Histology	Outcome
Koen et al. [5]	1998	5/Male	Increased stumbling and progressive bladder dysfunction	Lipomyelomeningocele	-	Mature	-
Hamada et al. [6]	2001	5/Male	Asymptomatic	Spina bifida	Subtotal	Mature	No change
Işik et al. [7]	2008	5/Female	Paraparesis	Syringomyelia	Total	Mature	Improved
Oktay et al. [2]	2016	12/Male	Back pain, lower limbs weakness, sphincter disorder	None	Subtotal	Mature	Improved
Keykhosravi et al. [1]	2020	12/Female	Back pain, progressive gait impairment	None	Total	Mature	Improved
Our case	2025	12/Female	Back pain, lower limbs weakness, sphincter disorder	None	Subtotal	Mature	Improved

Histologically, spinal teratomas are classified into the following three categories: mature, immature, and malignant [13]. Mature teratomas contain well-differentiated tissue, including chondrocytes, squamous epithelial cells, endocrine structures, mucosal cells, and neural elements, whereas immature teratomas typically exhibit fetal tissue and tend to have a more aggressive biological behavior. Malignant teratomas contain a malignant germ cell component and are more likely to recur or metastasize [1,2,13]. Because teratomas can contain both benign and malignant components within the same lesion, comprehensive histological examination of the entire specimen is essential [10,13].

Etiopathogenesis

The exact pathogenesis of spinal teratomas remains unclear. Two primary theories have been proposed to explain their origin. The misplaced germ cell theory suggests that primordial germ cells are aberrantly located along the midline during their migration from the yolk sac to the gonadal ridges, leading to teratoma formation [9]. The dysembryogenic theory postulates that teratomas are non-neoplastic lesions resulting from disorganized differentiation of pluripotent embryonic cells within the caudal cell mass [5]. The frequent association of spinal teratomas with congenital spinal abnormalities such as tethered cord syndrome, split cord malformation, scoliosis, and spina bifida supports the dysembryogenic theory [5,8]. However, in cases like ours, where no spinal dysraphism or congenital anomalies were observed, the misplaced germ cell theory appears more plausible.

Clinical presentation

The symptoms of intraspinal teratomas are typically non-specific and primarily depend on the tumor’s location and mass effect. Most patients present with progressive spinal cord compression, leading to neurological deficits [4,8,14]. When located in the conus medullaris, these tumors can cause nerve root compression or cauda equina syndrome [2,8,14]. Clinical manifestations may include limb weakness, back and/or leg pain, sensory disturbances, sphincter dysfunction, and, in some cases, sexual dysfunction [3,8]. Due to their varied presentation, spinal teratomas can mimic other space-occupying lesions. In rare cases, tumor rupture can lead to complications such as chemical meningitis, obstructive hydrocephalus, seizures, cerebral vasospasm, coma, or even sudden death [8,11].

Imaging

MRI is the imaging modality of choice for diagnosing spinal teratomas [1]. It provides essential information about the tumor’s location, size, and extent [1,8]. Typically, teratomas are well-encapsulated, thick-walled tumors with a circular or lobulated shape that may contain both solid and cystic components. They often exhibit heterogeneous signal intensity due to the presence of various tissue types, including fat and calcifications. Fatty tissue within the lesion usually exhibits hyperintense signals on T1- and T2-weighted images, and hypointense signals on fat-suppressed sequences. Calcified components are frequently present and generally appear hypointense across all imaging sequences. After administration of gadolinium-based contrast agents, these lesions tend to show only mild enhancement. Additionally, MRI can help detect associated congenital spinal anomalies such as spinal dysraphism [4,11,15]. Diffusion tensor imaging and tractography may assist in evaluating the tumor’s relationship with the spinal cord’s white matter tracts and aid in surgical planning [3]. However, MRI alone cannot always definitively differentiate teratomas from other intramedullary tumors such as ependymomas, astrocytomas, epidermoid cysts, dermoid cysts, or neurenteric cysts. Therefore, histopathological examination remains the definitive method for diagnosis [1,2,13].

Treatment and prognosis

Surgical resection is the treatment of choice for pediatric intraspinal teratomas, the goal being complete tumor excision whenever possible [1-4,8,11,16]. However, as demonstrated in this case, spinal teratomas can be strongly adherent to the conus medullaris and cauda equina nerve roots. In such instances, attempting a complete resection may pose a high risk of neurological injury, making subtotal resection a reasonable alternative. For tumor remnants that are densely attached to the spinal cord, low-power bipolar electrocoagulation may be used instead of direct excision to deactivate residual tumor tissue [8]. The optimal surgical approach should balance maximal tumor removal with the preservation of neurological function. Notably, recurrence rates for total and subtotal resection do not significantly differ (9% vs. 11%) [1,3,4,8].

Intraoperative neurophysiological monitoring (IONM) can significantly enhance surgical safety by enabling real-time assessment of spinal cord function [17]. However, this modality was not available at our institution. Notably, Chen et al. [8] reported a low complication rate in a cohort of 39 patients with conus medullaris teratoma who underwent surgery without the use of IONM [8]. Nevertheless, several studies strongly advocate for the application of IONM in spinal cord tumor surgeries, as it facilitates more extensive tumor resection while minimizing the risk of neurological deficits [17,18].

Although laminoplasty was not performed in this case, it may be preferable in selected patients, particularly children, to minimize postoperative spinal instability and dural sac compression due to epidural scarring [1,8].

Recurrence rates are influenced by both tumor histopathology and the extent of resection [1,2,8]. Mature teratomas have lower recurrence rates, even after incomplete removal [1-3,8]. Immature and malignant teratomas exhibit a higher risk of recurrence and require long-term follow-up with serial imaging [1,2].

Given the rarity of spinal teratomas, the role of adjuvant therapy remains unclear. While adjuvant radiotherapy may be considered for tumors with malignant components, chemotherapy is generally not recommended [19]. Further research is needed to determine the optimal management strategies for residual or recurrent tumors [8,11].

## Conclusions

Conus medullaris teratomas are rare entities, predominantly affecting adults and seldom seen in children. Their clinical and imaging features are non-specific, often mimicking other spinal cord tumors. While complete surgical resection remains the treatment of choice, subtotal resection may be a valid alternative when total excision poses a high risk of neurological damage, as demonstrated in our case. The role of adjuvant treatments in managing residual tumors remains uncertain and requires further investigation.
